# Supplementary material for: Characterizing cell-type spatial relationships across length scales in spatially resolved omics data
Source: Nat Commun. 2025 Jan 3;16:350. doi: 10.1038/s41467-024-55700-1 (PMC11699133; doi:10.1038/s41467-024-55700-1)
Supplement: Supplementary file 3 — Supplementary Software 1 [file 41467_2024_55700_MOESM3_ESM.zip › CRAWDAD/plots/slideseq/S4_slideseq_mli2_cooccurance.pdf]

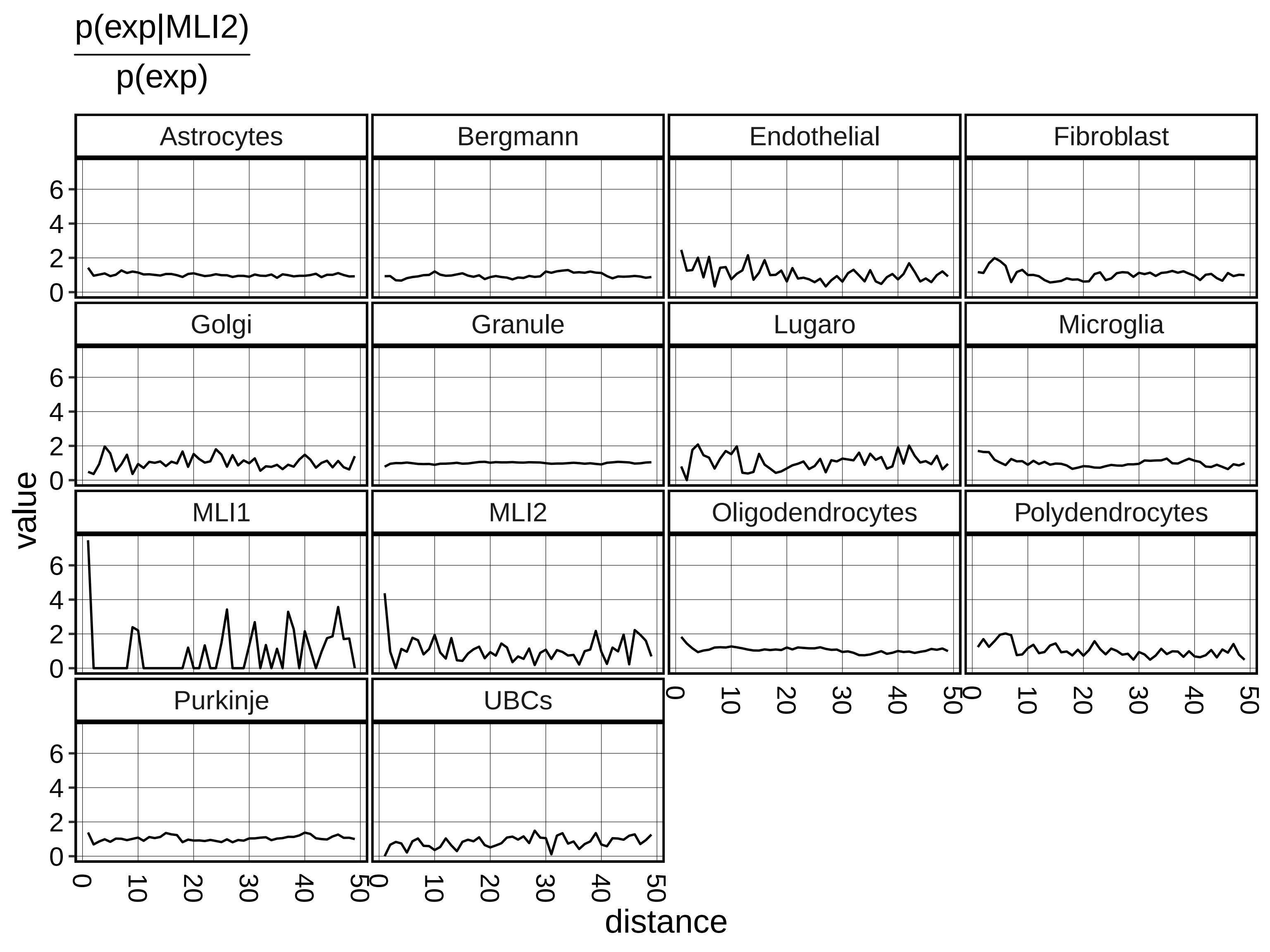

p(exp|MLI2) / p(exp)
Astrocytes
Bergmann
Endothelial
Fibroblast
Golgi
Granule
Lugaro
Microglia
MLI1
MLI2
Oligodendrocytes
Polydendrocytes
Purkinje
UBCs
value
distance
0
2
4
6
0
10
20
30
40
50
